# Reviewing the interface of bioeconomy and ecosystem service research

**DOI:** 10.1007/s13280-020-01374-0

**Published:** 2020-10-12

**Authors:** Dalia D’Amato, Bartosz Bartkowski, Nils Droste

**Affiliations:** 1 Helsinki Institute of Sustainability Science - Department of Forest Sciences, Faculty of Agriculture and Forestry, University of Helsinki, Helsinki, Finland; 2 Department of Economics, Helmholtz Centre for Environmental Research - UFZ, Leipzig, Germany; 3 Department of Political Science, Lund University, Lund, Sweden

**Keywords:** Agroecology, Biotechnology, Circular bioeconomy, Green economy, Natural capital, Sustainability transformations

## Abstract

The bioeconomy is currently being globally promoted as a sustainability avenue involving several societal actors. While the bioeconomy is broadly about the substitution of fossil resources with bio-based ones, three main (competing or complementary) bioeconomy visions are emerging in scientific literature: resource, biotechnology, and agroecology. The implementation of one or more of these visions into strategies implies changes to land use and thus ecosystem services delivery, with notable trade-offs. This review aims to explore the interdisciplinary space at the interface of these two concepts. We reviewed scientific publications explicitly referring to bioeconomy and ecosystem services in their title, abstract, or keywords, with 45 documents identified as relevant. The literature appeared to be emerging and fragmented but eight themes were discernible (in order of decreasing occurrence frequency in the literature): a. technical and economic feasibility of biomass extraction and use; b. potential and challenges of the bioeconomy; c. frameworks and tools; d. sustainability of bio-based processes, products, and services; e. environmental sustainability of the bioeconomy; f. governance of the bioeconomy; g. biosecurity; h. bioremediation. Approximately half of the documents aligned to a resource vision of the bioeconomy, with emphasis on biomass production. Agroecology and biotechnology visions were less frequently found, but multiple visions generally tended to occur in each document. The discussion highlights gaps in the current research on the topic and argues for communication between the ecosystem services and bioeconomy communities to forward both research areas in the context of sustainability science.

**Electronic supplementary material:**

The online version of this article (10.1007/s13280-020-01374-0) contains supplementary material, which is available to authorized users.

## Introduction

The bioeconomy is currently a driving concept at the policy and industry levels, promising to reconcile environmental and social goals with economic development, particularly with the renewal and prosperity of various primary and secondary sectors (El-Chichakli et al. 2016; Dietz et al. 2018). Broadly speaking, the bioeconomy promotes resources derived from living biomass as alternatives to fossil resources, with knowledge and innovation being key enablers of such a shift. However, multiple understandings and definitions of the bioeconomy have been advanced in policy and science (Bugge et al. 2016; Dietz et al. 2018; Holmgren et al. 2020).

Several countries worldwide have adopted bioeconomy strategies, with different foci (Dietz et al. 2018). National strategies in Europe differ according to domestic biomass availability in each country, but they generally rely on the contribution and development of a wide range of economic sectors and industries, including forestry, food, chemistry, pharmaceuticals, and textiles (Hoff et al. 2018; Bruckner et al. 2019). On the other hand, the strategy forwarded by the United States strongly emphasizes biofuels and biotechnology (Staffas et al. 2013; De Besi and McCormick 2015; Bracco et al. 2018). Overall, the international political discourse on bioeconomy strongly focuses on the economic dimension, while environmental and social considerations are accessory (Kleinschmit et al. 2017; Ramcilovic-Suominen and Pülzl 2018).

Pfau et al. (2014) noted that scholars hold diverse perspectives regarding the contribution of the bioeconomy to sustainability, with the sustainable sourcing of biomass being a crucial issue. They concluded that while a positive contribution to sustainability is not self-evident, it should be the main goal of a bioeconomy. They called for inter- and transdisciplinary research as the key to enabling a sustainable bioeconomy. Similar concerns have been raised by several other authors (e.g. El-Chichakli et al. 2016; Kröger and Raitio 2017). Some principles for environmental sustainability are being increasingly integrated within a comprehensive definition of bioeconomy. These include the socially and environmentally sustainable sourcing of biomass and the cascading use of biomass. The latter, drawing from the waste hierarchy principle of the circular economy, advocates for the reuse and recycling of resources by prioritizing high-value biomass uses before energy use^1^ (Ciccarese et al. 2014; Bezama 2016).

The Global Bioeconomy Summit (2018) has defined the bioeconomy as a transformative process that contributes to the Sustainable Development Goals. However, multiple Sustainable Development Goals, including lower-level targets, are not necessarily always compatible (Weitz et al. 2018; Schaafsma and Bartkowski 2020). In other words, trade-offs occur when economic, social, and environmental goals are pursued simultaneously, especially considering a strong sustainability perspective in the long term. An inclusive conceptualization of the bioeconomy, along with suitable metrics, is needed to monitor the progress of national and international policies in line with broader sustainability ideals such as the Sustainable Development Goals (Hák et al. 2016; Wolfslehner et al. 2016; Karvonen et al. 2017).

Notably, current discussions on the conceptualization and implementation of the bioeconomy have not yet been strongly linked to the ecosystem services concept, i.e. a mainstream concept currently advanced in academia and policymaking for sustainable land use. Pfau et al. (2014, p. 1240) suggested that ‘[a]n ecosystem services perspective may provide a useful framework to consider the use of biomass resources for various goals, provided that utilization is realized within the boundaries of sustainability’. Since becoming mainstream in international research and policymaking at the beginning of the millennium (MA 2005; TEEB 2010b), the ecosystem services concept has served as a pivotal reference framework for conceptualizing and operationalizing sustainability transformations. Its main prerogative is to highlight the relevance of natural capital and the contribution of ecological processes to human well-being (Braat and de Groot 2012). Importantly, the ecosystem service framework allows for the identification and analysis of synergies and trade-offs between various societal objectives and impact dimensions (Cord et al. 2017; Schaafsma and Bartkowski 2020). It has been applied in various contexts relevant for the bioeconomy, including multifunctional agriculture (e.g. Albert et al. 2017; Palomo-Campesino et al.^2018^), forestry (e.g. Makkonen et al. 2015), urban systems (e.g. Gómez-Baggethun and Barton 2013), and marine ecosystems (Hattam et al. 2015). Moreover, the ecosystem service concept has increasingly been adopted in policies and decision-making (Bouwma et al. 2018).

As both lines of research – the bioeconomy and ecosystem services – address the environmental and social sustainability of resources and land use, work at their interface represents an important space and a fruitful avenue forward. Marchetti et al. (2015), Hetemäki et al. (2017), and Székács (2017) proposed interesting reflections concerning the bioeconomy and its tensions with natural capital and other environmental and ecological aspects. Recently, Palahí et al. (2020) have advocated for a biodiversity-based circular bioeconomy, with integrated solutions that enable equitable and participatory transformations of industrial sectors, urban areas, and land-food-health systems. Overall, however, we know little about the interface of the bioeconomy and ecosystem services research communities. In this article, we address this research gap through a comprehensive review of the literature. In particular, the review aims to explore the interdisciplinary space between the bioeconomy and ecosystem services concepts by analysing the literature that makes explicit use of both terms. This narrow search strategy allows focusing on the conscious interactions occurring between the two research fields. Against this background, we assess: (i) the publication trend in time; (ii) the geographical location and ecosystem under study; (iii) the methods employed in the study; (iv) the bibliographic links between documents; (v) the themes emerging from the documents; (vi). the bioeconomy products and other ecosystem services relevant in the study; (vii) the bioeconomy vision(s) implicitly or explicitly adopted by the studies; viii. the relation between themes and bioeconomy visions.

The rest of the manuscript is organized as follows. In the ‘Conceptual background’ section, we provide a conceptual reference linking the ecosystem services and the bioeconomy as ideas and research fields contextual to sustainability. We describe methods and results in the respective sections. In the ‘Discussion’ and ‘Conclusions’ sections, we discuss the results in the context of sustainability transformations and outline conclusions and recommendations relevant for researchers and other professionals.

## Conceptual background

### Ecosystem services as a sustainability concept

The ecosystem services framework has been used internationally to assess and govern the socio-economic values (and underlying tensions) of various ecosystems and biomes, with analyses at global and local scales (TEEB 2010a, 2011). The concept and its applications have survived and evolved after complex self-reflection processes mainly regarding the utilitarian framing (Droste et al. 2018), with extensive discussion over the theoretical and technical limitations of ecosystem services assessment methods and indicators (e.g. Cord et al. 2017).

The ecosystem services idea generally relates to a strong sustainability vision, where social and economic activities are fundamentally dependent on the biophysical system (Folke et al. 2016). The relationship between natural and human systems is depicted as a closed loop (Haines-Young and Potschin 2010): ecological processes useful to humans are defined as ecosystem services; these contribute to various dimensions of human well-being, including basic material needs, security, physical and psychological health, and social cohesion.

Ecosystem services depend on the historical, geographical, and socio-economic context of individuals or groups of beneficiaries (Oteros-Rozas et al. 2014). Ecosystems are thus managed according to the underlying values that society wants to pursue. The implicit choices behind various types of ecosystem management inevitably produce synergies or trade-offs between multiple ecosystem services and between sustainability dimensions (economic, societal, and environmental goals) (Braat and de Groot 2012; Smith et al. 2017).

Strategic ecosystem management may thus positively and/or negatively affect various individuals or groups of beneficiaries (or stakeholders) (Davies et al. 2015). Importantly, the source of such effects may be remote in space and time (Schröter et al. 2018). Public–private dynamics are central to this tension, where environmental benefits are often privatized and costs are public (Bartkowski et al. 2018).

Part of the ecosystem services research focuses on developing and improving biophysical and socio-economic assessment methods and indicators to measure the above-mentioned complex dynamics (Müller and Burkhard 2012; Costanza et al. 2017). Importantly, drivers of synergies and trade-offs for ecosystem services are only occasionally explicitly taken into account by scholarly analyses (Dade et al. 2019). Driver include e.g. land-use and natural resource management, and related policy changes. In this context, bioeconomy strategies may be understood as an important background to such drivers.

### Bioeconomy visions as sustainability strategies

Three bioeconomy visions emerge from the analysis of scientific literature and policy documents (Bugge et al. 2016; Hausknost et al. 2017; Meyer 2017; Priefer et al. 2017; Vainio et al. 2019).

*The resource vision* emphasizes substituting fossil resources-based products with bio-based products through research, development, and technological innovation and the establishment of new value chains capitalizing the conversion of biomass into new products (Bugge et al. 2016). This substantially means favouring the upgrading of biological raw materials. Nonetheless, the resource vision remains resource intensive. The biological resources needed are procured from land and water biomass, especially involving the primary production sectors, such as forestry and agriculture but also fisheries.

*The biotechnology vision* regards the application and commercialization of scientific and technological development, especially leveraging (often proprietary) knowledge from life sciences and bioresources (cf. Bugge et al. 2016; Meyer 2017). This vision is characterized by more ‘disruptive and radical innovations’ compared to the resource-oriented bioeconomy (Bugge et al. 2016, p. 10). Examples include health applications, such as personalized medicine, biopharmaceuticals, and biocosmetics (Bugge et al. 2016); increasing crop productivity by means of engineered diversification or improvement (i.e. genetic modification); biopesticides, biofertilisers, biostimulants, and bio-based chemicals and materials that require less environmentally burdening processing compared to traditional alternatives (Lokko et al. 2018).

*The agroecology vision* is concerned with enhancing integrated, multifunctional land management leveraging rural/territorial knowledge, social innovation, and socio-ecological resilience (Hausknost et al. 2017). This occurs through solutions, such as scaling up of traditional ecological practices in agricultural systems to minimize external inputs while relying on and enhancing natural processes such as synergies of diversified crops (Altieri et al. 2012). Moreover, the idea exists of re-connecting people and material/energy flows between urban and rural systems (Bugge et al. 2016). This includes e.g. ‘the use of own waste as well as waste from urban areas […] to reduce or even eliminate the need for external inputs to bioproduct production facilities’ (Bugge et al. 2016, p. 12). Food security, diversity, and sovereignty are central elements in this vision, especially in emerging economies (Marsden and Farioli 2015; Pereira et al. 2018).

**Fig. 1:**
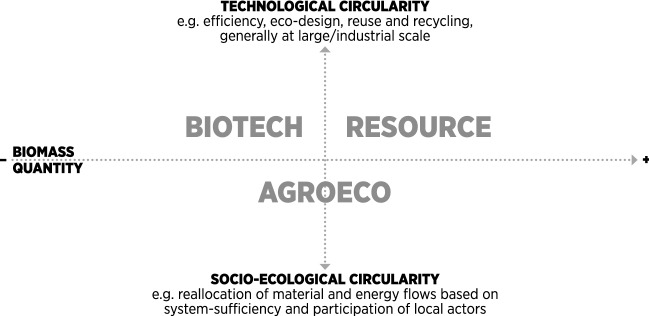
The three bioeconomy visions in relation to biomass requirements and circularity type. *Source*: Own representation

We suggest that rather than conflicting, these visions may be interpreted as overlapping or complementary. We also propose that they may be classified according to two dimensions: i) the amount of biomass appropriated in societal throughput; and ii) the type of innovation or solution proposed (Fig. 1). Resource and biotechnology visions are more oriented towards ‘technological fixes’ in the industrial utilization of bio-based resources (focus on large-scale, centralized solutions from a global and regional partnership).^2^ Largely drawing from the circular economy (Kirchherr et al. 2017; Korhonen et al. 2018a, b), technological fixes are generally intended for large or industrial scales, include efficiency, eco-design, reuse, and recycling,^3^ and biotechnology advancement is additionally emphasized (Meyer 2017). The agroecology vision is more oriented towards a logic of socio-ecological innovation, with a re-allocation of material and energy flows within a system boundary oriented towards sufficiency and participation of local actors, generally at a smaller scale). According to Hausknost et al. (2017) and Meyer (2017), the latter vision emerges from scientific and societal debates, while the first two visions are present in the official bioeconomy strategies.

### Linking ecosystem services, bioeconomy, and sustainability

Because of its immediate metaphorical power and the flexibility of its toolbox, the ecosystem services concept has shown potential in bridging various academic disciplines and societal groups and contributing to fostering sustainability (Abson et al. 2014; Droste et al. 2018; Steger et al. 2018). We argue that ecosystem services thinking may also be relevant for monitoring the implementation of bioeconomy strategies by accounting for trade-offs and helping to assess multidimensional problems in land use and beyond (see Karvonen et al., 2017; Bruckner et al. 2019). To establish such a link, we draw from the idea of impacts and dependencies of human activities on ecosystem services in social-ecological systems (D’Amato et al. 2018, 2020).

Generally, the bioeconomy is characterized by a dependency on provisioning services, specifically biomass but also genetic resources and information-based ecosystem services (i.e. biosecurity, bioprospecting, cultural services related to education and scientific advancement). As such, all bioeconomy visions fundamentally rely on the supply of (various) ecosystem services while affecting the supply through land and resource management. In fact, as many ecosystem services are co-produced, bioeconomy visions and related strategies/actions inform the management of socio-ecological systems.

In the following, we outline our reflections concerning the dependencies and impacts of each vision. The bioeconomy resource vision typically entails land-use intensification, which would affect certain regulating services (especially climate regulation, soil and water quality, and pollination) and cultural services such as recreational and aesthetic values (Smith et al. 2017; Gasparatos et al. 2018b). The biotechnology vision would imply a more limited demand for biomass but stronger dependence on genetic resources, whereas the agroecology vision combines a dependency on biomass and genetic resources along with regulating and cultural services. The agroecology vision appears to be the only one that emphasizes the multifunctionality of ecosystems and thus offers a more holistic perspective on the full spectrum of ecosystem services.

A specialization of urban and rural area functions and associated ecosystem services seem to be at the centre of the resource vision at the local and global levels (with trade-offs at risk of being exported to emerging or developing economies). In the biotechnology vision, relevant stakeholders are particularly individuals or groups upholding territorial or scientific knowledge and genetic resources (with risks associated with privatization and appropriation). The agroecology vision entails a reconnection of rural and urban communities (with a risk of inequity through functional regionalization).

**Fig. 2:**
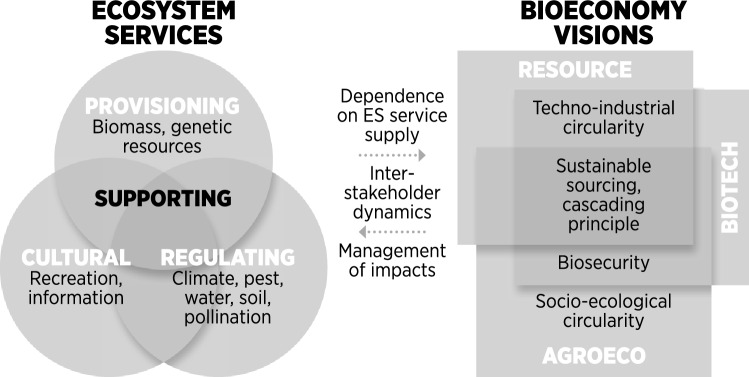
Conceptual framework of the relations between circular bioeconomy visions and ecosystem services

The framework in Fig. 2 brings together the current understanding of the three main bioeconomy visions and how they relate to ecosystem services through the impacts and dependencies that affect ecosystem services and the related stakeholder groups at local and global scales. Such dynamics affect human well-being and consequently societal values, ultimately informing management and governance of natural capital. The framework reproduces the logic proposed by the ecosystem services cascade framework (Haines-Young and Potschin 2010; Braat and de Groot 2012) to link natural capital and ecosystem services to human values (and vice versa, Leviston et al. 2018).

## Methods

Our study represents a comprehensive, systematically structured review of the scientific literature (following suggestions on how to organize a review by e.g. Livoreil et al. 2017). Scopus and Web of Science were used as search engines in March 2019. In Scopus, we used the following search string: TITLE-ABS-KEY (("bioeconomy" OR "bio economy" OR "bio-economy" OR "biobased economy" OR "bio-based economy" OR "bio based economy") AND ("ecosystem service*" OR "environmental service*" OR "natural capital" OR "Nature's Contributions to People")). In Web of Science we used the following search string: WOS: TS = (“bioeconomy” OR “bio economy” OR “bio-economy” OR “biobased economy” OR “bio-based economy” OR “bio based economy”) AND TS = (“ecosystem service*” OR "environmental service*" OR "natural capital" OR "Nature's Contributions to People"). Our search incorporated publications for all years, and we applied our search to the title, abstract, and authors’ keywords for Scopus and to the title, abstract, authors’ keywords, and keywords plus^4^ for Web of Science.

We are aware that this search strategy excluded some scholarly research on issues tangentially related to the bioeconomy or ecosystem services. For example, we signal research on biofuels (Gasparatos et al. 2018a, 2018b) and on the North–South perspective of global bioeconomy value chains (Virchow et al. 2014; Scheiterle et al. 2018). We outline the motivation for our search strategy in the following.

We did not include search terms tangentially related to bioeconomy and ecosystem services because we wanted to exclusively target the documents that explicitly used the terms ‘bioeconomy’ (rather than, “biofuels” or “bio-based products”) and ‘ecosystem services’ (rather than, “natural resources”) as background concepts for their research. Our assumption is that those authors had consciously adopted the bioeconomy and ecosystem services ‘conceptual package’, lens, or perspective (see ‘Conceptual background’ section) in developing their article. Given our arguments in ‘Conceptual background’ section, showing the implicit relatedness of the two concepts, this search strategy was in line with our intent to target research that links the two fields in an explicit and conscious way.

We only searched the title, abstract, and keywords because we wanted to collect documents that consistently referred to the bioeconomy and ecosystem services. In fact, previous literature (Abson et al. 2014; Droste et al. 2018) has noted that the word ‘ecosystem services’ may be used as a buzzword in parts of the scientific literature and the relation to the concept may not necessarily be deeply articulated. We suspect this may also be the case for ‘bioeconomy’.

Our search resulted in 42 publications from Scopus and 50 from Web of Science, which were further screened to a. remove double entries (31); b. exclude book chapters; c. exclude documents with full text in languages other than English; d. exclude documents not relevant for this review: for example, documents where the concepts of bioeconomy or ecosystem services (or their synonyms) were not sufficiently reported in the full text (i.e. mentioned ≤ 1 time) or documents dealing with bioeconomic modelling^5^). The final sample thus included 45 publications.

We thoroughly read through the documents collected, and extracted the following information from the full text of each article: the method(s) used, and the geographical region and ecosystem under study. Furthermore, we took note of which bioeconomy product(s) and other ecosystem services (classified using the CICES 5.1 groups, Haines-Young and Potschin (2018)) along with trade-offs and synergies were mentioned in the abstract. This choice was motivated by the difficulty in determining which bioeconomy products or ecosystem services were mentioned in the full text. Often, the documents would only vaguely mention an item, despite it not really being the focus of the research. Thus we relied on the abstract to determine which bioeconomy products and ecosystem services were central in the study.

This information was used to compile a descriptive overview of the literature (‘ Overview of the literature retrieved’ section). Basic bibliographic analysis (bibliographic maps) was performed by means of the VOSviewer software (van Eck and Waltman 2010), with bibliographic data from Scopus as input. VOSviewer connects related items (e.g. papers or country affiliations), which are depicted as circles whose size reflects a relevant magnitude (e.g. number of citations of a paper or number of authors within an affiliation) according to metrics such as co-authorships or shared citations. For details of the algorithms used to generate the graphs, see van Eck and Waltman (2010).

Two independent thematization steps were additionally performed based on analysis of the full texts (results presented in ‘Emerging themes’ and ‘Relation to bioeconomy visions’ sections, respectively). In the first thematization step, each author independently categorized each document into one of eight themes, identified through an inductive process where the data. The categorizations were then compared and a common categorization agreed upon. In the second thematization step, the authors independently classified each document into one or more of the three bioeconomy visions previously identified in the scientific literature, assuming that a vision was clearly identifiable (see ‘Bioeconomy visions as sustainability strategies’ section). This second process was thus deductive, where the categories (i.e. bioeconomy visions) were pre-selected based on the conceptual background. The entire analysis, including the collection of descriptive information and the first and second steps of thematization were initially performed independently by each author, who then discussed the process together to reach a consensus between at least two authors (out of three).

## Results

### Overview of the literature retrieved

**Fig. 3:**
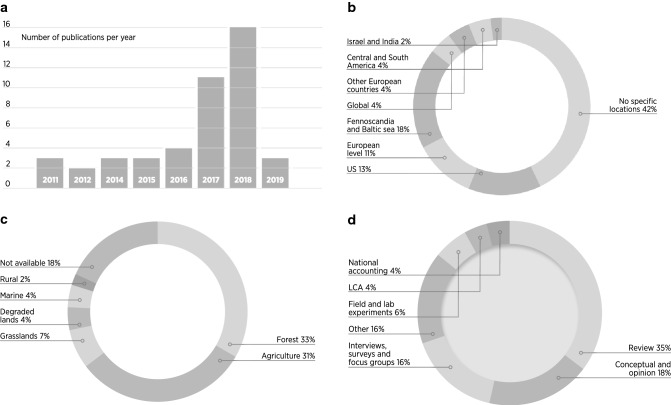
Overview of the reviewed documents, including (clockwise): publication trend in time; geographical location of study; methods used; ecosystem studied. *Source*: Own representation

The 45 documents selected for review (see Electronic Supplementary Materials S1 for a full overview of the data) were published between 2011 and 2019, with a positive trend in publication rate over the years (Fig. 3). The reviewed documents were either published in scientific journals or conference proceedings. Journals included specialized outlets on biotechnology or bioenergy, forestry, and agricultural journals, along with more broad sustainability-related journals (e.g. Ecosystem Services, Journal of Cleaner Production). Less than half of the studies had a specific geographic focus, ranging from individual countries to geographic or political regions (e.g. the Baltic area, Europe). Most documents dealt with a specific type of ecosystem, mostly forest (33%) and agricultural systems (31%). One-third of the articles dealing with forests were based in Finland or Norway. Reviews, conceptual analyses, and opinion articles were the most frequent methods employed in the documents.^6^ Several reviews were not systematic assessments of the existing literature, but rather loose reviews. Qualitative research methods, such as interviews, surveys, and focus groups, represented 16% of the document.

**Fig. 4:**
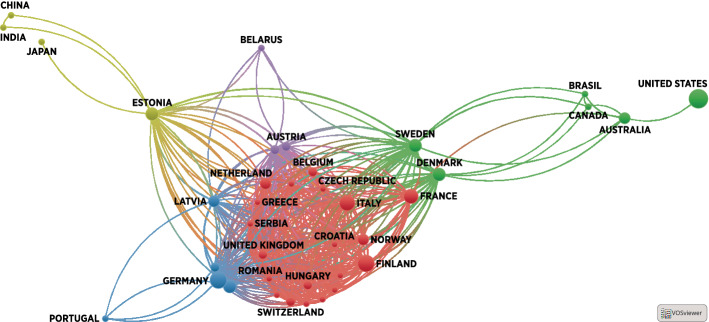
Bibliographic analysis of authors’ affiliations by country (circle size indicates the number of authors for each affiliation node, while links indicate co-authorship relations). *Own representation*

**Fig. 5:**
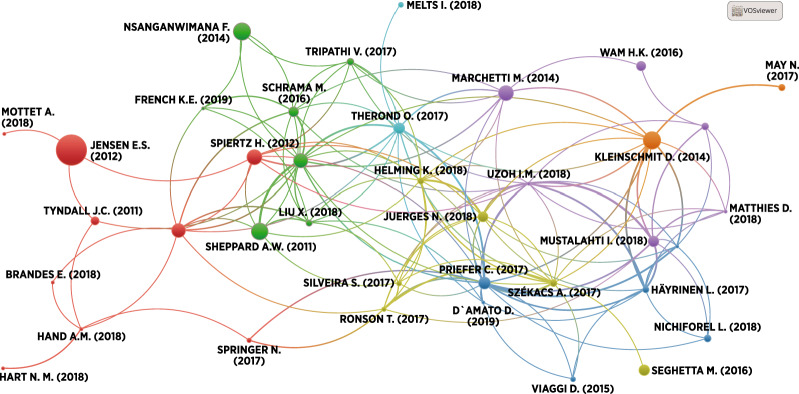
Bibliographic coupling between the reviewed documents (circle size indicates the number of overall citations, while links indicate shared citations within the sample). *Own representation*

In terms of authorship, the majority of the affiliations were from the United States (nine documents), Germany, and Finland (seven documents each). Bibliographic analysis of co-authorship by affiliation (Fig. 4) shows the existence of large, strongly interconnected European clusters, which have only a few links to non-European authors, especially to the United States (Fig. 4, note that circle size indicates the number of authors for each affiliation node, while the links indicate co-authorship relations). Figure 5 depicts the bibliographic coupling (shared citations) occurring between the studies included in our review. Quite substantial overlap occurs in the sources cited by the documents (note that circle size indicates the number of overall citations, while links indicate shared citations within the sample).

In accordance with the observation above, i.e. that many studies focused on the bioeconomy, generally only half of the studies specified some bioeconomy product: less than half specified an ecosystem service in the abstract (conversely, certain studies specified more than one bioeconomy product or ecosystem service) (Table 1). In the 25 studies mentioning bioeconomy products, 40% of the items were related to the production and use of biomass in general; for example, Tyndall et al. (2011) studied the perceptions of foresters on woody biomass potentials, while Ronzon and Piotrowski (2017) looked at the relevance of agricultural residues as a general resource for the bioeconomy. Bioenergy was mentioned in 31% of the cases, i.e. biogas for electricity generation or heating, in terms of suitability of particular feedstocks (Nsanganwimana et al. 2014) or the effects of first- and second-generation bioenergy crops on soils (Schrama et al. 2016). Furthermore, 14% of the specified bioeconomy products were biofuels, 6% were food, and 3% each of biomaterials and fertiliser.

**Table 1:** Bioeconomy products and other ecosystem services present in the abstracts of the reviewed documents. Note that abstracts occasionally mentioned more than one product or service. *Own representation*

Documents	Share (relative to the total number of documents)	Bioeconomy products	Share (relative to the total number of products) (%)
Mentioning bioeconomy products	56%	Biomass	40
		Bioenergy	31
		Biofuel	14
		Food	6
		Biomaterials	3
		Fertilizers	3

		Ecosystem services	Share (relative to the total number of services) (%)
Mentioning ecosystem services	36%	Lifecycle maintenance, habitat and gene pool protection	20
		Regulation of soil quality	17
		Mediation of wastes or toxic substances	10
		Water conditions	10
		Atmospheric composition and conditions	10
		Reared or wild plants and animals for nutrition, materials or energy	10
		Regulation of baseline flows and extreme events	7
		Pest and disease control	7
		Physical and experiential interactions with nature	7
		Wild plants for nutrition, materials or energy	3
		Wild animals for nutrition, materials or energy	3

The studies we reviewed incorporated a diverse range of ecosystem services. “Lifecycle maintenance, habitat and gene pool protection” was the most frequent CICES category, mentioned by 20% of the studies that explicitly referred to ecosystem services in their abstracts. References to this ecosystem service (mostly simply as “biodiversity”) range from rather vague and general (e.g. Vega and Madrigal 2017) to quite specific, e.g. rhizosphere biodiversity and its potential for the bioeconomy (Uzoh and Babalola 2018). Seventeen per cent of the cases referred to “Regulation of soil quality”, e.g. when looking at legumes as potential biofuel feedstocks (Jensen et al. 2012) or the impact of bioenergy feedstocks on soils (Schrama et al. 2016). “Mediation of wastes or toxic substances”, “Water conditions”, “Atmospheric composition and conditions”, and “Reared or wild plants and animals for nutrition, materials or energy” each have a 10% share of the specified ecosystem services, followed by “Regulation of baseline flows and extreme events”, “Pest and disease control”, and “Physical and experiential interactions with nature” (7% each).

We only found a few references to the concept of ecosystem service trade-offs and synergies. The term “trade-off(s)” can be found in six abstracts, although two cases use it in a fairly general manner. Eyvindson et al. (2018) explicitly investigated trade-offs between various forest ecosystem services and biodiversity in the context of the bioeconomy. Wam et al. (2016) also look at trade-offs between multiple ecosystem services, but the link to bioeconomy is not as pronounced in their study. Using the trade-off concept in a more specific context, Liu et al. (2018) look at carbon footprints and reactive nitrogen emissions related to various types of fuels, while Jensen et al. (2012) emphasize trade-offs arising between conventional uses of legumes and their use as biorefinery and biofuel feedstock. The term “synergies” is present in one single abstract only (Silveira et al. 2017) and is used in a rather general sense.

### Emerging themes

Eight themes were identified across the reviewed publications: Technical and economic feasibility of biomass extraction and use (N = 11); Potential and challenges of the bioeconomy (N = 9); Frameworks and tools (N = 7); Sustainability of bio-based processes, products, and services (N = 5); Environmental sustainability of the bioeconomy (N = 4); Governance of the bioeconomy (N = 4); Biosecurity (N = 3); and Bioremediation (N = 2).

#### Technical and economic feasibility of biomass

This is the most recurrent theme, dealing with the potential of biomass in terms of profitability, availability, or feasibility. Method-wise, the articles in this cluster are either based on statistics or on stakeholder perspectives. Overall, there is a relatively strong business perspective, including considerations regarding environmental and social benefits, such as enterprise diversification, maintenance of local biodiversity and ecosystem services, and valorization of marginally productive land and maintenance of ecosystem services. Mitchell et al. (2016), Brandes et al. (2018), and French (2019) investigated profitability and crop suitability issues in bioenergy production. Ronzon and Piotrowski (2017) and Springer et al. (2017) estimated biomass availability for bio-based material and bioenergy sectors, with a focus, respectively, on the European Union and the United States Midwest. Such estimates entail implications in regional planning in terms of ecosystem services-related sustainability issues. Häyrinen et al. (2017) and Tyndall et al. (2011), Hand and Tyndall (2018), and Hart et al. (2018) investigated agricultural and forest stakeholder opinions on the feasibility and potential avenues of the bioeconomy in natural resource management and use. Jensen et al. (2012) highlighted the potential of legumes in the development of agrosystems to reconcile biomass needs and ecosystem services maintenance, especially in regard to climate change mitigation under a bioeconomy. Uzoh and Babalola (2018) discussed the role of biotechnology in enhancing rhizosphere biodiversity for soil fertility under a resource-intensive bioeconomy.

#### Potential and challenges of the bioeconomy

This is the second most recurrent theme and includes macro-level or conceptual analyses, sometimes focusing on a specific geographic region or economic sector. Papers in this cluster express the need for further and more sophisticated assessments of changes in land use and ecosystem services, integrating all value dimensions (ecological, economic, and socio-cultural) and for coordinated and participatory decision-making processes, especially regarding ecosystem services-related policies. Concepts in this cluster included the bio- and circular economy, entrepreneurship and eco-innovation, and sustainability. Life Cycle Analysis (LCA) is a method and tool for measuring the impacts of research. Székács (2017) broadly challenged the idea of the bioeconomy being sustainable by definition and discussed environmental and social aspects, including ecosystem services and planetary boundaries. On a similar note, Marchetti et al. (2015) provided points of reflection to address the tension between bioeconomy implementation and the maintenance of natural capital and ecosystem services in the forest sector context. Kleinschmit et al. (2014) and Priefer et al. (2017) reflected on the bioeconomy as a discourse, elaborating on environmental and social themes and on the potential contribution of social sciences to the development of the bioeconomy in the forest sector. According to Viaggi (2015), emerging phenomena may be of interest for enriching and renewing the notion of productivity, used to evaluate research impacts in agriculture. Mansuy (2015) provided a brief overview of the potentials and challenges of acquiring and sharing socio-economic and environmental big data in the forest sector context. Sasson and Malpica (2018), and Vega and Madrigal (2017) reviewed the state-of-the-art and potential development of the bioeconomy in Latin and Central America, respectively. Silveira et al. (2017) discussed bioenergy initiatives and systems in eight Baltic countries, including the link between bioeconomy and ecosystem services. Strategic planning is recommended to mitigate trade-offs and enhance synergies with ecosystem services.

#### Frameworks and tools addressing bioeconomy challenges

These are largely intended as conceptual instruments for assessment or governance purposes. Trade-off assessments in particular are argued to be important. Conflicts between stakeholders may damage environmental sustainability and socio-economic value creation and are thus detrimental to an articulated goal of the bioeconomy. Therefore, trade-offs between competing interests on ecosystem management or use must be recognized, assessed, and managed. Angelstam et al. (2019) used a toolbox of ‘diagnosis and treatment’ for sustainable landscapes to assess social-ecological systems in forests on the east and west parts of the European continent. Helming et al. (2018) presented an impact assessment framework linking bioeconomy-driven societal targets and related strategies to soil functions and ecosystem services (and vice versa). They argued that the bioeconomy would likely require increased production, while measures are needed to monitor and minimize stress on soils. Coupling interviews with stakeholders and algae cultivation, Ingle et al. (2018) proposed a framework for integrated pest management in seaweed farming in the context of a marine bioeconomy. Magnus and Magnus (2015) offered a ‘dialogical method’ for assessing the outcomes of interventions supporting sustainable development in rural areas, in regard to natural, social, human, cultural, political, economic, and built capital. Through a literature review, Therond et al. (2017) created a classification of farming systems and agricultural models to address sustainability issues in the industrial agriculture context. Six models were identified based on the levels of biological vs. chemical inputs and on the level of territorial vs. globalized dynamics. While discussing ecological effects of biofuel production in terrestrial systems, Raghu et al. (2011, p. 15) proposed a framework of biocomplexity, capturing ‘multidimensional and cross-disciplinary considerations of biofuels production’. Wam et al. (2016) proposed a model for evaluating ecosystem services trade-offs by including monetary and non-monetary information.

#### Sustainability of bio-based processes, products, and services

The studies within this theme assess or discuss sustainability aspects at various analysis levels of production and consumption systems, often with regard to biofuels. Trade-offs may be observed in the production of most fuels, but biofuels from low-input farming or waste as feedstock have the smallest carbon and nitrogen footprints. Through a systematic review and content analysis of the literature, May et al. (2017) identified the available environmental indicators and methods for assessing wood products sustainability along the entire life cycle. Liu et al. (2018) performed a comparative analysis of carbon and nitrogen emissions from fossil fuels and biofuels (first, second, and third generation). Seghetta et al. (2016) unpacked sustainability issues of a potential case of seaweed production and biorefining, finding synergies with water purification and climate change mitigation. Stahel (2017) briefly provided an overview of the Circular Economy Package by the European Commission, remarking that the package also takes elements of natural capital (water, bioeconomy) into consideration. D’Amato et al. (2019) performed a content analysis of reports by 123 companies operating in land-use intensive sectors, revealing that while circular economy practices are consistently reported across all companies and sectors, references to green and especially bioeconomy practices are numerically under-represented in the reports.

#### Environmental sustainability of the bioeconomy at the land-use level

The documents included in this theme examine the environmental and ecological effects of implementing resource-intensive bioeconomy strategies. Research in this theme deals with management approaches such as landscape-scale strategies to minimize environmental impact. As (environmental) trade-offs are based on societal values, stakeholder acceptability does come into play in this cluster. Eyvindson et al. (2018) looked at the effects of increasing forest harvest levels on biodiversity and non-timber ecosystem services under a bioeconomy strategy. Matthies et al. (2018) elicited the perspective of environmental students on bioeconomy-related forest management in Finland, revealing that the acceptance of various ecosystem management objectives by individuals is influenced by perceived harms and benefits, along with their gender and knowledge on the topic. Melts et al. (2018) reviewed literature concerning the long‐term effects of fertilisation in semi-natural grasslands; they argued that negative effects occur on community biodiversity and productivity, and on the overall stability of ecosystem services. Schrama et al. (2016) studied the effects of first- and second‐generation bioenergy crops on soil quality, concluding that the choice of bioenergy crops have a great positive or negative influence on the maintenance of soil ecosystem services, also affecting future crops.

#### Governance of bioeconomy

The documents dealing with these themes addressed disparate governance issues that may foster or hamper implementation of the bioeconomy. Based on the opinion of governmental and non-governmental forest stakeholders, Aurenhammer et al. (2018) investigated preferences regarding the most appropriate societal institutions and instruments for solving disputes over forestland use. A comprehensive review conducted by Juerges and Hansjürgens (2018) of institutions and instruments in soil governance suggested that this research area is undeveloped despite pressures on soil resources being likely to increase in the transition towards a bioeconomy. Mustalahti (2018) discussed the need for citizen participation and inclusion in the current bioeconomy discourses in the context of the forest sector in Finland. Nichiforel et al. (2018) analysed property rights in European forests, showing large variations in private landowners’ freedom in deciding about their management goals and use, and in limiting others from using forest resources.

#### Biosecurity

This theme relates to food security along with the management of pests, diseases, and new entities. It deals with uncertainty, risk management strategies, and with concrete threat assessments. For example, Sheppard et al. (2011) suggested that a bioeconomy-driven increase in novel crops, especially through the use of biotechnology, may represent a threat to current and future production systems, biodiversity, ecosystem services, and human health. Identified risks included allergens and toxicity issues, and the uncontrolled spread of abandoned trial cultivations and invasive species. Mottet et al. (2018) discussed the role of domestic herbivores in food security, in conjunction with considerations on climate change, rural livelihoods, and gender issues. Spiertz (2012) reviewed the role of agronomy in food security to meet the multiple demands of the bioeconomy, calling for improvements at the crop, farm, and landscape levels to achieve food security, sustainability, and ecosystem services at regional and global scales.

#### Bioremediation

This is an environmental restoration technique employing microorganisms and/or other organisms to revert pollution and other forms of degradation. Nsanganwimana et al. (2014) assessed the suitability of *Miscanthus* as a suitable crop for combining biomass production and ecological restoration of contaminated and marginal lands. Tripathi et al. (2017) argued that biotechnological advancements hold important potential for context-specific, additional interventions to restore degraded land for multiple ecosystem services, including biomass production.

### Relation to bioeconomy visions

**Fig. 6:**
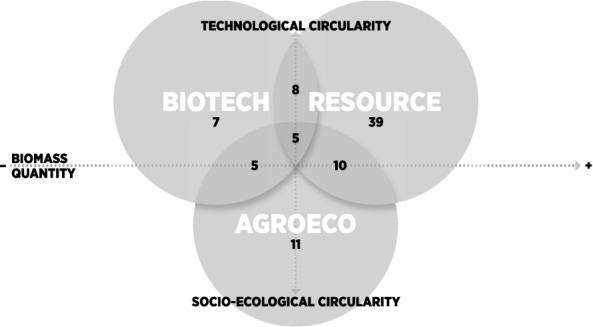
Occurrence of bioeconomy visions in the reviewed documents, based on the qualitative understanding of the authors after reading the literature each document could contain more than one vision). *Own representation*

We analysed which, if any, bioeconomy vision was present in the reviewed literature. We allowed each document to potentially adopt more than one vision of the three visions identified in the conceptual background section: resource, agroecology, biotechnology. Of the 45 documents included in this review, 40 dealt with at least one bioeconomy vision, while five documents had no discernible relationship to a specific vision. The resource vision had the largest share and was also the most diffuse (Fig. 6). Resource-oriented documents focused on biomass, bioenergy, and biofuels in forest systems, and on the use of biological resources in general. Substantial overlap of the resource vision occurred with the other two, and the agroecological vision was coupled with the resource vision in all but one case. Thus, the resource vision was dominant across all recorded documents. Regarding the interaction with themes, the resource vision was mainly associated with the themes *Technical and economic feasibility of biomass*, *Potential and challenges of the bioeconomy*, and *Sustainability of bio-based processes, products, and services*. These publications have a clear focus on how biomass may be grown in a profitable yet sustainable manner.

**Fig. 7:**
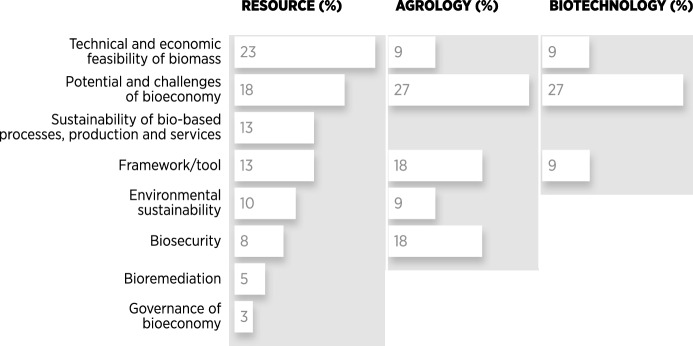
Distribution of themes for each of the three visions, expressed as per cents. *Own representation*

Agroecology was the second most prevalent vision. Documents linked to this vision mainly dealt with agricultural systems, for example in regard to organic production, ecological pest management, or ecological restoration of soils and agroecosystems. A substantial overlap occurred with the other two visions: one agroecology document also presented a resource vision and approximately half of the documents also associated with the biotech vision. While emphasizing the ecological side of agricultural production, this vision was thus not mutually exclusive with either a resource-based or a biotechnological perspective. *Potential and challenges of bioeconomy*, *Environmental sustainability*, and *Biosecurity* were the main themes of the documents linked to the agroecology vision (Fig. 7). Thus, there was a focus on the environmental side of bio-based production processes.

The biotechnology vision contained the smallest number of documents. Examples included research concerning breeding programmes, genetically engineered organisms, and phytoremediation. All documents in this vision had also been classified into the resource vision and more than half into both the resource and the agroecology visions. Biotechnology perspectives thus included ecological processes in production. *Potential and challenges of the bioeconomy* and *Technical and economic feasibility of biomass* were the main themes linked to the biotechnology vision. Here, the emphasis was on the corresponding challenges in biotechnological production.

In summary, the three visions often overlapped and were by far not mutually exclusive. Resource vision was the most prevalent, followed by agroecology and biotechnology. Where the resource vision places an emphasis on biomass production (and subsequent energetic uses), the agroecology vision stresses the environmental side of production and biotechnology deals with genetic and cellular processes. Overall, only a handful of documents integrate all three perspectives, but a large degree of integration occurs across the themes.

## Discussion

The literature linking the bioeconomy and ecosystem services concepts is an emerging niche showing an increasing trend in terms of documents per year. This is in line with both the momentum recently gained by bioeconomy literature (D’Amato et al. 2017) and the continuing upwards trend exhibited by the ecosystem services literature over the past 10–15 years (Droste et al. 2018). As they represent two key concepts in sustainability science and policymaking, it seems logical and somehow inevitable that the bioeconomy and ecosystem services must come to be discussed together, especially in terms of the effects that the implementation of bioeconomy strategies have on ecosystem services. Nonetheless, the scientific articles considered relevant for this review were few (45), and thus the intersection of the two disciplines proved greatly disconnected and fragmented, with very few documents drawing equally from both concepts.

Based on the authors’ affiliations and the geographic focus of the documents, the literature stems from the United States and Europe (especially Fennoscandia) and is mostly applied in that context, with solid collaboration between individual European countries. The discussion concerning the role and opportunities of developing and emerging economy countries in the bioeconomy was therefore largely missing (see Hoff et al. 2018), despite being central to the global and long-term sustainability of the bioeconomy.

Forest and agricultural systems represent dominant ecosystems at study, as these are key providers of ecosystem services for the bioeconomy (Ollikainen 2014; Bugge et al. 2016). An evident gap is the lack of studies on aquatic, and particularly marine, ecosystems which are often believed to harbour large opportunities for the bioeconomy in terms of efficient biomass provisioning (e.g. Scarlat et al. 2015). Only two studies in our review focused on marine ecosystems.

The most common theme regarded the *Technical and economic feasibility of biomass extraction and use*, including punctual and context-specific studies concerning biomass production. The second most frequent theme, *i.e. Potential and challenges of the bioeconomy*, included global- or macro area-level studies discussing the potential and overall challenges of the bioeconomy, especially regarding trade-offs between bioeconomy-related economic and social goals with environmental ones. Several *Frameworks and analytical tools* were proposed in the reviewed literature, providing instruments for addressing sustainability issues— including ecosystem services— in the context of bioeconomy-driven strategies or goals. These studies draw from existing ideas, frameworks, or classifications such as Integrated Pest Management (IPM), driver-pressure-state-impact-response (DPSIR), and ecosystem services classification and valuation (respectively, see e.g. Ehler 2006; Gabrielsen and Bosch 2003; MA 2005). A certain potential therefore exists for the generalizability and adaptation of such instruments beyond the context examined in those publications. Moreover, we signal a concept/tool that did not emerge in our review, but that is of interest in the context of national competitiveness in the bioeconomy: the biomass value web accounts for interlinkages of value chains deriving from the cascading use of biomass (Scheiterle et al. 2018). This idea is leveraged in developing and emerging economies (Virchow et al. 2014) to highlight bioeconomy-related avenues for such countries ‘to go beyond their role of pure raw material suppliers’ (p. 16) and ‘to generate jobs and income in the biomass producing, processing and trading sector, particularly in rural areas’ (p. 18).

In regard to *sustainability assessment of bio-based products and processes*, certain scholars have noted that LCA methods fail to capture sustainability issues related to ecosystem services, especially at the land-use level (Rugani et al. 2019; D’Amato et al. 2020). This is increasingly important in assessing the sustainability of bioeconomy products and processes, which fundamentally rely on and impact land use.

Other emerging themes included the *Environmental and social sustainability of the bioeconomy at land-use level*, *Governance issues, Biosecurity,* and *Bioremediation. S*tudies thematized under governance mostly treated the topic in a general manner. While markets are seen to have an important role, the state is expected to secure the provision of ecosystem services, in contrast to calls for future markets to regulate this field. The possibility of landowners and other stakeholders accessing and benefitting from ecosystem services is an important issue (see also Bartkowski et al. 2018). More specific analyses are still lacking e.g. concerning land-use or agri-environmental policy instruments, such as payments for ecosystem services, in the context of bioeconomy. Beyond the need to understand the trade-offs involved in advancing the bioeconomy, there is an increasing need for instruments that will help ensure its sustainability.

The methods used in the reviewed studies reveal a certain degree of multidisciplinarity in this field. Notably, the most commonly used empirical methods represent qualitative research approaches to eliciting stakeholder perspectives (e.g. young professionals, agricultural and natural resources stakeholders). These methods may contribute to investigating the issue of acceptability regarding the bioeconomy, which has been raised as a critical point in forwarding the bioeconomy (e.g. Ollikainen 2014; Meyer 2017; Korhonen et al. 2018a, b; D’Amato et al. 2019).

Acceptability issues in the bioeconomy extend beyond the social appreciation of bio-based products or bioeconomy-related ecosystem services by consumers (e.g. Toppinen et al. 2013; Hori et al. 2019) and also include—more broadly—the legitimacy of bioeconomy conceptualization, and goals and measures as understood by experts and the public. Examples of contested issues in this context include sustainable biomass sourcing, property rights and appropriation of bioresources, genetic information or traditional knowledge, along with the use and perceptions of genetically modified organisms (Chapotin and Wolt 2007; Pfau et al. 2014).

The acceptability of the politically driven bioeconomy agenda is thus worth exploring among various societal actors. Nonetheless, as noted by Karvonen et al. (2017, p. 3), ‘acceptance may not guarantee sustainability if, for example, general acceptance leads to the consideration of only short-term benefits while neglecting long-term perspectives’. It is thus striking that only a few studies have used the ecosystem service toolbox to ascertain or manage the net sustainability contribution of the bioeconomy, which is likely to have numerous conflicting ecological and social impacts. The few studies applying LCA are a notable exception to this, though they usually focus on particular products. Based on the reviewed documents, trade-off analyses are central but often not operationalized in terms of ecosystem services (Cord et al. 2017). Similarly missing are land-use models linking bioeconomy with multifunctional landscapes (Verburg et al. 2016; Castro et al. 2018). Footprint analyses, such as the one by Bruckner et al. (2019), would likely benefit from being complemented by ecosystem service information. One option would be to use ecosystem service-based scenario analysis to investigate possible bioeconomy futures (cf. Hagemann et al. 2016; Rosa et al. 2017).

Overall, the literature dealing with the bioeconomy and ecosystem services tends to adopt either a resource vision of bioeconomy or a mixture of the three visions (resource, biotechnology, and/or agroecology). Note that visions are generally not explicitly acknowledged in the literature. The resource vision is more often associated with forest systems, while the agroecology vision is found in studies dealing with agricultural systems, also reflecting the origin and tradition of this concept. Principles from the agroecology vision may potentially be relevant to forest systems, and this may be worth investigating further. The relative dominance of the resource vision is not particularly surprising, as, in its essence, the use of biotic resources is the bioeconomy’s central concept. Nonetheless, the scarcity of documents in the scientific literature that engage with the biotechnology vision is puzzling, given that the interpretation for a bioeconomy agenda by the Organization for Economic Cooperation and Development (OECD) focuses on biotechnologies.

Ultimately, the tension between how and which biological resources are used remains the central node in the relationship of bioeconomy–ecosystem services (e.g. low- vs. high-value products; technological vs. social innovation) (Brunori 2013). The multiplicity of ideas and voices is already evident in the three visions identified in the bioeconomy community, as discussed in  ‘Bioeconomy visions as sustainability strategies’ and ‘Linking ecosystem services, bioeconomy, and sustainability’ sections. However, such tensions reach beyond the discussion of trade-offs between bioeconomy-driven economic, social, and environmental goals. One key contribution of the ecosystem services concept to sustainability science is the attempted shift from a resource-centred logic towards an understanding of the human economy and society as embedded within planetary natural boundaries (Folke et al. 2016). The new idea of the bioeconomy appears to be rather focused on resources, circling back to a more traditional—and according to certain scholars, anachronistic—vision of human–nature relations. It is noteworthy to signal recent attempts in scientific (e.g. Pfau et al. 2014; Liobikiene et al. 2019) and policy- or industry-level documents (e.g. EC 2018; Global Bioeconomy Summit 2018) to acknowledge and reconcile the bioeconomy’s conflicts with other social and ecological goals. In this context, we argue that an ecosystem services perspective as an increasingly integrative concept (see Droste et al. 2018) may contribute to further easing strong sustainability and long-term temporal perspectives into the shaping of bioeconomy strategies while also providing insights for practical assessment tools (see also Gasparatos et al. 2018a, 2018b).

## Conclusions

Our study reviewed publications dealing with the links between the bioeconomy and ecosystem services, both increasingly central concepts in sustainability science and decision-making. While a shared research ground is only emerging, communication between the ecosystem services and the  bioeconomy communities holds potential, especially in relation to the following issues.

1. The conceptualization of the human–nature relations proposed by the ecosystem services community—i.e. shifting the focus from natural resources to services and human well-being—may represent an important element in further advancing the conceptual development of the currently strongly resource-centred bioeconomy.
2. Frameworks, tools, indicators and data developed in the context of ecosystem services research may be valuable for addressing sustainability issues related to various bioeconomy strategies, including synergies and trade-offs between and within sustainability dimensions. Existing data and instruments from the ecosystem services research may also be integrated with assessment methods already in use (e.g. LCA).

Future direction for researchers and other professionals working at the interface of the two concepts should include the assessment of ecosystem services synergies and trade-offs occurring under various bioeconomy strategies, or in other words, the impacts of the bioeconomy on multiple ecosystem services. This may contribute to addressing the acceptability and legitimacy of issues that are central to sustainability science and practice concerning e.g. the sustainable sourcing of biomass or the use and manipulation of bioresources.

## Electronic supplementary material

Below is the link to the electronic supplementary material.

Supplementary file1 (XLSX 5682 kb)

## Footnotes

1 Note that cascading entails a series of technical and practical limitations (Toppinen et al. 2020). In addition, requirements for learning and innovation in cascading might entail certain initial costs, in terms of substitution of natural capital for human and other capital, in order to achieve stronger sustainability solutions over a long-term time frame.

2 In this context, the concept of a circular bioeconomy has recently been advanced to address the sustainability limitations affecting the bioeconomy alone (Antikainen et al. 2017; Hetemäki et al. 2017). The development of this concept is at an embryonic stage despite being mentioned in scientific and policy documents.

3 See e.g. the cascading use of biomass (drawing from the waste hierarchy principle), advocating for the reuse and recycling of resources in a logic that prioritizes high-value uses of biomass before energy use (Ciccarese et al. 2014; Bezama 2016).

4 Web of Science differs from Scopus in that it applies the search also to a set of keywords that are not provided by the authors but are instead generated by the search engine based on an algorithm. These additional keywords consist of words and phrases harvested from the titles of the cited articles.

5 Bioeconomic models are economic-ecological models used to inform management of natural resources. Despite the homonymous etymology, bioeconomic models do not relate to bioeconomy as a political strategy or development vision.

6 Each document may employ more than one method.
